# Induction of labour: creation of a classification of Grenoble allowing an assessment of the evaluation of practices

**DOI:** 10.1186/s12884-022-04487-4

**Published:** 2022-02-21

**Authors:** Manon Vanneaux, Pierre-Louis Forey, Véronique Equy, Pascale Hoffmann, Didier Riethmuller

**Affiliations:** Département de Gynécologie-Obstétrique & Médecine de la Reproduction, Centre Hospitalier Universitaire Grenoble Alpes (CHUGA), La Tronche, France

**Keywords:** Induction of labour, Cervical ripening, Classification, Pregnancy

## Abstract

**Background:**

Induction of labour, a very common obstetric procedure, affects about one in five pregnant women in most developed countries. Induction of labour is medically indicated, is subject to risks and additional costs, and is often poorly experienced by patients. The practices concerning induction vary widely from centre to centre and therefore need to be evaluated. Our aim was to develop a tool for evaluating induction of labour which would facilitate geographical and temporal comparisons.

**Methods:**

We have created a classification based on the principles of the internationally known Robson classification. It should be simple, robust, reproducible and require readily available data in each file. The groups are fully inclusive and mutually exclusive. This classification has been validated by a Delphi method.

**Results:**

Our classification includes 8 clinically relevant groups according to 5 obstetrical criteria. In order to classify each patient into a group, a simple system based on a maximum of 7 successive questions (from 1 to 7 questions) is used. Our classification has been validated by 13 national experts with satisfactory overall approval.

**Conclusions:**

With a view to improving the quality of care, our Grenoble classification would allow a standardization of the evaluation of practices of the induction of labour over time in the same maternity hospital. It would also allow the comparison of practices within different maternity hospitals in a network, a country or even different countries.

## Background

The evaluation of modern obstetric practices is based on the analysis of different indicators associated with quality of care. This evaluation involves the use of tools designed to classify situations and analyse practices. Most of the time, these tools correspond to classifications.

The induction of labour (IOL) is undoubtedly one of these indicators. To date, it is one of the most common obstetric interventions and affects at least one in five pregnant women in most developed countries.

IOL is subject to indications and, like any medical intervention, to risks and additional costs. Induction practices currently vary greatly from one maternity hospital to another and it seems essential to evaluate them in order to make better use of this medicalised approach.

Even if there is no consensus on the “right” labour induction rate, there is undoubtedly an interest for each maternity hospital to compare its practices over time and geographically with those of other maternity hospitals at the level of a network, and then nationally and even internationally.

The Nippita classification described in 2015 proposed a new classification of IOL [1]. However, compared to Robson classification, it was not disseminated internationally despite being more than 5 years old and does not represent a reference tool for assessing practices in each center.

## Methods

Our objective was to create a tool for assessing the IOL on the principles of Robson classification [2]. This internationally known classification facilitates the evaluation of caesarean section practices. This new evaluation tool would allow analysis of the induction rates in each maternity hospital and facilitate geographical and temporal comparisons. It does not represent an induction score according to the indications of induction.

Our classification is created on the basis of predefined obstetrical criteria. It must be simple to use, robust and require data that is readily available in each patient’s medical file. Furthermore, it must also be reproducible with low inter-operator variability.

The number of groups should be sufficient to give details on practices but limited so as not to lose the overall picture. The order and relationships between groups are also important to allow for quick and easy interpretation of the data.

The different groups in the classification should reflect, as far as possible, the most relevant groups of patients in clinical practice. These groups should be fully inclusive, meaning that each patient should be able to be placed in one of the groups, and mutually exclusive, meaning that each patient should only be able to be placed in one of the groups.

We have validated our Grenoble classification by a Delphi method [3]. This is a structured interactive technique for developing a consensus or near-consensus among experts on what to include in a study or a tool such as our classification.

The experts fill in a questionnaire anonymously and then receive feedback on all responses from the entire panel of experts. The questionnaire is revised based on these responses and returned to the panel. This process is repeated until the range of expert responses narrows sufficiently to build consensus or near consensus on all or some of the issues. In each round, the experts can send comments and suggestions. A Delphi survey is conducted with a group of people who are considered to have professional expertise in the field under study, in our case obstetrics.

For our classification, an email invitation was sent to 13 experts. They had to have more than five years of clinical experience and had to be actively practicing in the field. They had to practice their profession independently and were ignorant to each other.

Those who responded to this invitation and agreed to participate in all phases of the Delphi process were considered to have provided written and informed consent and were included in the committee.

In the first questionnaire, the experts were asked to judge the clinical relevance of each group in our classification according to a Likert scale with 5 response choices (“not at all relevant”, “not very relevant”, “no opinion”, “rather relevant” and “very relevant”). They were also asked to explain their choice if they answered “not at all relevant” or “not very relevant”.

In the second questionnaire, changes were made to the classification according to the comments of the first round and explanations were given to all experts.

Each group was considered validated if more than half of the experts gave a “very relevant” or “rather relevant” answer.

## Results

Our classification is based on 5 obstetrical criteria present in all medical records: the number of fetuses (single pregnancy or multiple pregnancies), the presentation of the fetus (cephalic or breech), the gestational age in weeks, the existence of prelabour rupture of membranes (PROM) at term, the existence of a maternal or fetal pathology.

**Fig. 1:**
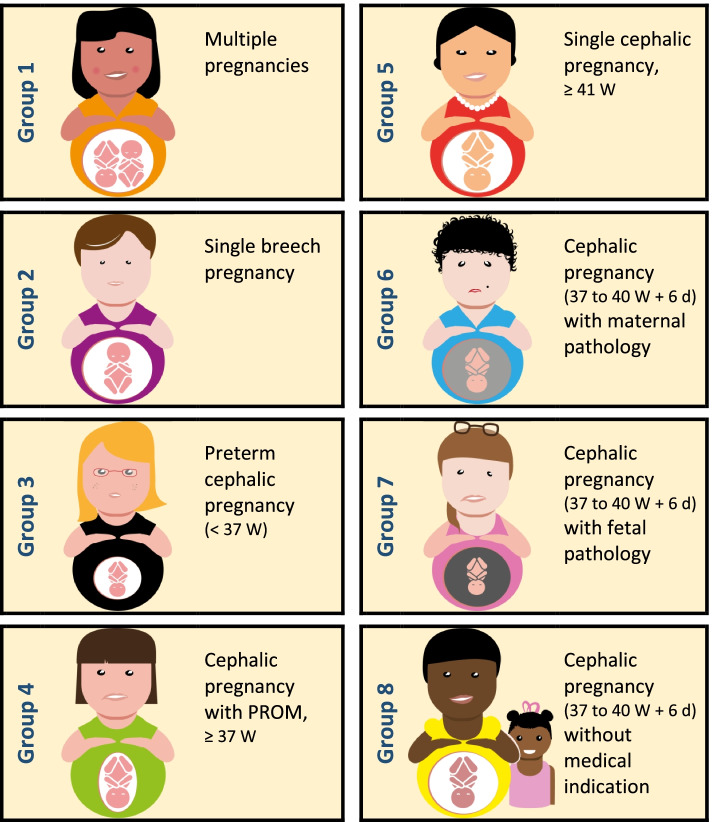
The Grenoble classification of artificial induction of labour into 8 groups

It is composed of 8 groups considered clinically relevant, which are as follows (Fig. 1):

- Group 1: Multiple pregnancies,
- Group 2: Single breech pregnancy,
- Group 3: Preterm single cephalic pregnancy (term less than 37 weeks gestation),
- Group 4: Single cephalic pregnancy with prelabour rupture of membranes at term (37 weeks gestation and above),
- Group 5: Single cephalic pregnancy, late term and post term (41 weeks gestation and over), intact membranes,
- Group 6: Single cephalic pregnancy, term between 37 and 40 weeks, 6 days with maternal pathology indicating induction, intact membranes,
- Group 7: Single cephalic pregnancy, term between 37 and 40 weeks, 6 days with fetal pathology indicating induction, intact membranes,
- Group 8: Single cephalic pregnancy, term between 37 and 40 weeks, 6 days, induced without medical indication, intact membranes.

The groups single cephalic pregnancy, late term and post term (group 5) and single cephalic pregnancy with PROM at term (group 4), each account for a significant proportion of the inductions.

The groups late term and post term (group 5) and preterm (group 3), represent specific situations particularly risky.

In the preterm single cephalic pregnancy (group 3), maternal dysgravidia and fetal pathologies are often inseparable, justifying the creation of a single group.

As the groups comprising the inductions between 37 and 40 weeks, 6 days (group 6 to 8) represent a significant proportion of the inductions, we thought it would be interesting to classify the patients concerned according to the indication of the induction.

Concerning the groups 6 and 7, the allocation of each patient to one of the groups does not depend on the severity of the condition for which she is classified. It is not considered that a maternal pathology has priority over a fetal pathology. In case of maternal and fetal pathology in the same patient, it is necessary to know whether the maternal pathology is responsible for the fetal pathology. If this is not the case, we need to define which pathology makes the decision to induce. For example, in the case of a patient with severe pre-eclampsia and intrauterine growth retardation, the maternal pathology is responsible for the fetal picture and the patient belong to group 6. On the other hand, in the case of intrauterine growth retardation without pre-eclampsia, the patient belong to group 7.

The group of inductions without medical indication is an increasingly important part of this practice and varies greatly from one maternity hospital to another.

The single breech pregnancy (group 2) and multiple pregnancies (group 1) represent a small number of the population studied. However, their particularities did not allow them to be included in the other groups.

**Fig. 2:**
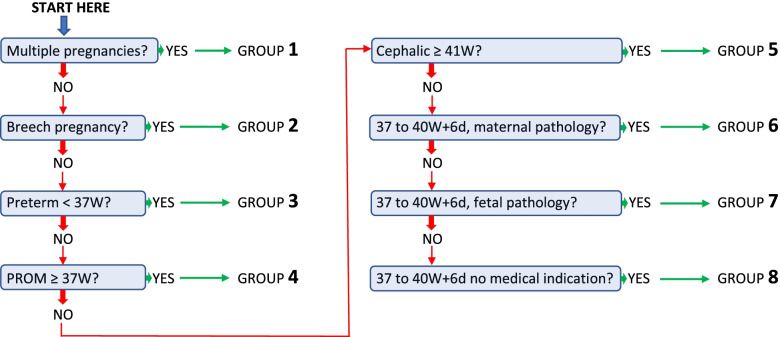
Simple system to classify each patient in one of the groups of the Grenoble classification

In order to classify each patient in her group, it is necessary to use a simple system based on a maximum of 7 successive questions (Fig. 2). The order of the groups has been defined according to the order of the questions to be asked. For each question, if the answer is positive, the patient is placed in the corresponding group. Conversely, if the answer is negative, the process is continued until a positive answer is given to one of the questions. If all the questions are answered in the negative, the patient is placed in group 8.

The different groups in the classification reflect the most relevant groups of patients in clinical practice, concerning the IOL. The analysis of the different groups will make it possible to target those representing significant part of the IOL in each center. However, this initial analysis will probably not explain the problems for the groups concerned. For this, other intra-group analyses may be performed depending on additional obstetric factors (for example, number of previous pregnancies, history of caesarean section …). Characteristics such as parity and previous caesarean section are not relevant in creating the 8 groups of our classification. Indeed, they are not factors involved in the decision to induce labour but possibly in the method used, which is not analysed by the classification.

Our classification has been validated using a Delphi method. We solicited 13 nationally recognised experts in obstetrics working in several French departments. A consensus was quickly reached for each group after only 2 rounds of the Delphi method. Following the first round, according to the experts opinion, we modified the titles of group 8 and group 4. Group 8 was changed from “induction of labour by convenience or psychological indication” to “induction without medical indication”. Group 4 has been changed from “premature rupture of membranes at term” to “prelabour rupture of membranes at term”, according to the recommendations established by the French national college of obstetricians (CNGOF). These modifications were validated during the 2nd round with explanations.

The overall approval of our classification was very satisfactory. Indeed, 4 groups were judged “rather relevant” or “very relevant” by 13 experts, 2 groups by 12 experts and 2 groups by 9 experts. Thus, each group could be validated. No group was judged “not at all relevant”.

We conducted a retrospective study evaluating our labour induction practices using our classification. The aim was to compare our practices between the year 2017 and 2019 within the CHUGA maternity unit. Our induction rate has decreased from 26.7 to 21.1% between 2017 and 2019. In both year, 4 groups are predominant. These are group 4 (30.8% in 2017 versus 30% in 2019), group 5 (18.9% versus 28.5%), group 6 (22.2% versus 20%) and group 7 (14.2% versus 12%). These results allow us to work on certain predominant groups such as groups 4 and 5 in order to improve the quality of our care.

## Discussion

The IOL is one of the most common obstetric procedures. Several authors have carried out an overview necessary to update knowledge on induction practices, particularly in France [4, 5]. However, there is no recognised and widespread assessment tool for this practice in the literature. It seems essential to be able to create a system for monitoring and comparing induction rates within institutions and between populations, analysing trends over time and comparing differences in maternal and perinatal outcomes. It can also be used for clinical benchmarking and performance measures of clinical quality.

Nippita et al. have proposed classification system to categorize women undergoing labour induction [1, 6, 7]. It was not disseminated internationally despite being more than 5 years old. It described 10 groups to assess labour induction practices. The clinical relevance of some groups is questionable. For example, the value of separating inductions of labour between 37 and 38 weeks gestation from those between 39 and 40 weeks gestation since they represent equivalent risks. Or the value of taking into account parity and previous caesarean section in some groups. On the contrary, IOL without medical indication does not appear although it is an increasingly frequent practice. The same is true for PROM at term which account for a significant proportion of IOL.

Influenced by Robson classification [8], we have developed a new tool to assess the practices of the IOL in the form of a classification that results in 8 mutually exclusive and fully inclusive groups. Using 5 readily available and reliably collected variables as the basis of the classification system, these proposed groups are clinically sound, simple, clear and easy to implement in a consistent manner at institutional, regional, national and international levels.

All the groups in our classification can be further subdivided and some groups can be merged to provide more appropriate denominators based on the events and results analysed. In addition, intra-group analyses can be carried out for those representing a significant proportion of the inductions in each centre, taking into account additional obstetrical factors if necessary.

In our Grenoble classification, we take into account indications of inductions in certain groups. This may therefore lead to a bias in interpretation [9]. However, these are large classes of indications without the need to go into diagnostic detail. The risks of inter-operator variability and bias are thus greatly minimised.

Our classification is the only one to have been validated by thirteen national experts using a Delphi survey [3]. The Delphi survey is an iterative, multi-stage process designed to transform opinion into group consensus [10]. The overall approval of each group was very satisfactory, allowing their validation after only 2 rounds of questionnaires. From the outset, we proposed to the experts our classification with the groups defined in advance. One of the alternatives could have been to ask the experts about the initial obstetrical criteria in order to then gradually create the different groups.

Widespread use of this practice assessment tool would make it easier to compare local, regional and international rates of IOL. It should improve our ability to compare homogeneous populations of women, thereby contributing to improved quality of care.

We have already used our classification to analyse the practices of IOL in our maternity in 2017 and 2019. This study allowed us to target groups representing a significant proportion of IOL. Thus, we can carry out further studies on these clinical situations to improve the quality of our care and then monitor the evolution of our practices over time using our classification. It would of course be very interesting to be able to compare the results of our different groups with other maternity hospitals.

## Conclusion

We have created a tool for assessing the IOL practices that would allow analysis of induction rates in each maternity ward and facilitate geographical and temporal comparisons. This tool corresponds to a simple, robust classification of 8 groups based on 5 obstetrical criteria that are readily available in each patient’s chart. It has been validated by a Delphi method.

This work is part of an approach to improve the quality of care in obstetrics based on the principle of the collegial evaluation of the benefit-risk balance. It is now necessary to take into account patient demand without increasing the risk of feto-maternal morbidity and mortality, in order to improve the experience of childbirth whatever the mode of the beginning of labour.

## Data Availability

The datasets used and/or analysed during the current study are avalaible from the corresponding author on reasonable request.

## Abbreviations

PROM: Prelabour rupture of membranes
W: Weeks gestation
CNGOF: French national college of obstetricians
IOL: Induction of labour
